# Eating Disorders among College Students in France: Characteristics, Help-and Care-Seeking

**DOI:** 10.3390/ijerph17165914

**Published:** 2020-08-14

**Authors:** Marie-Pierre Tavolacci, Pierre Déchelotte, Joel Ladner

**Affiliations:** 1 CIC 1404, Rouen University Hospital, U 1073, Normandie Univ., UNIROUEN, F 76000 Rouen, France; 2 Department of Nutrition, Rouen University Hospital, U 1073, Normandie Univ., UNIROUEN, F 76000 Rouen, France; pierre.dechelotte@chu-rouen.fr; 3 Department of Epidemiology and Health Promotion, Rouen University Hospital, U 1073, Normandie Univ., UNIROUEN, F 76000 Rouen, France; joel.ladner@chu-rouen.fr

**Keywords:** university student, eating disorder, stress, healthcare, general practitioner

## Abstract

The aim of this paper was to identify the characteristics of broader categories of eating disorders (ED) and help- and care-seeking among college students. An online cross-sectional study was conducted among students of the University of Rouen-Normandy, France. The Expali-validated algorithmic tool, combining SCOFF test (Sick, Control, One stone, Fat, Food) and body mass index, was used to screen eating disorders into three diagnostic categories: restrictive eating disorders, bulimic eating disorders, and hyperphagic eating disorders. A total of 1493 college students were included; mean age was 20.1 years (SD = 1.9). The prevalence of likely cases of eating disorder was 24.8% (95% CI, 22.6–27.0). Percentage distributions of bulimic eating disorders, hyperphagic eating disorders, and restrictive eating disorders were 13.3%, 8.6%, and 2.9%, respectively. The two main resources for help-seeking in emotional stress situations were friends and family, whatever the ED. Students with eating disorders consulted their general practitioner more often for stress or anxiety than students without eating disorders: hyperphagic eating disorders (44.9%), restrictive eating disorders (35.1%), bulimic eating disorders (30.2%), and no eating disorder (20.4%) (*p* < 0.0001). The prevalence of healthcare renunciation was 21.9%, with a higher risk among students with bulimic eating disorders (AOR CI 95% 1.91 (1.34–2.72). The findings show one quarter of students screened positive for an eating disorder. Stress management was not necessarily different between students with eating disorders and students without eating disorders, but the former had a greater risk of renouncing treatment, especially related to a fear of seeing a general practitioner.

## 1. Introduction

Eating disorders (EDs), as anorexia nervosa, bulimia nervosa, and binge eating, are serious mental illnesses characterized by disturbances of body image and eating behavior [1]. A recent literature review indicated that binge eating, then bulimia nervosa and anorexia nervosa, had the highest lifetime prevalences [2]. College years coincide with the typical age of onset for EDs [3,4], and it is well-documented that EDs are a significant concern among college students [5,6] with a higher risk of restrictive EDs [7]. College years fall into a crucial developmental phase, known as “emerging adulthood” [8]. Academic life is an integral part of the life of all college students, and without a healthy attitude toward academic goals, students may undergo stress. The academic pressures of meeting grade requirements, test-taking, the volume of material to be learned, and a job in parallel have been shown to be a significant source of stress for students [9]. Academic stress is caused by high workloads, but also coincides with a stage of life in which students must face many changes; this situation causes a change in the habits of young people related to their practice of physical activity and food [10]. EDs in college students could be associated with lower academic performance [11], comorbid mental disorders, and somatic conditions [12]. Over 70% of individuals with EDs report comorbid disorders, such as anxiety disorders (>50%) and mood disorders (>40%) [12].

Like many young people, only about one quarter of college students with a mental health problem seek professional care [13]. Healthcare renunciation, that is, foregoing or delaying healthcare, has been a focus of public health debates in the past decade [14]. Several barriers have been identified which prevent students from seeking help for mental health problems, and are linked not only to financial constraints, but also to individuals’ subjective needs and ways of facing illness [15]. One study reports reasons for healthcare renunciation, such as lack of time, lack of perceived need, and a desire to deal with the issue “on my own” [16]. These other reasons for healthcare renunciation should be investigated among students with stress and anxiety.

The early detection and treatment of EDs is of key importance because of the life-threatening complications of EDs [17]. Fewer than 10% of cases of bulimia nervosa and binge-eating disorder and fewer than 50% of cases of anorexia nervosa are detected by general practitioners (GPs). Therefore, in order to simplify the diagnostic approach to primary care, diagnostic categories of ED have been proposed by some authors [18]. Patients with EDs often present emotional difficulties and some form of denial that may prevent them from seeking specific treatment from health care systems. There are also practical barriers, such as the cost of treatment or a lack of knowledge about help resources [19,20].

However, to our knowledge, no study has focused on college students transitioning into adulthood and EDs. The aim of this study was thus to investigate the characteristics of eating disorder diagnostic categories and help- and care-seeking among college students.

## 2. Methods

A cross-sectional study was conducted among students of the University of Rouen-Normandy, France in March 2018 and March 2019. Students were invited to participate in the study via the weekly news bulletin of the university emailed to the 30,000 students of Rouen-Normandy University and/or via information posted on our Facebook page, “Ta Santé en un Clic” (“Your Health in One Click”), with about 300 subscribers. Volunteer students filled in an anonymous online self-questionnaire via a link on the website (www.tasanteeunclic.org). Students over 25 years of age were excluded from the analysis. The observational study design was approved by the Commission Nationale de l’Informatique et des Libertés (the French Electronic Data Protection Authority) and by Rouen University Hospital’s Institutional Review Board without mandatory informed consent (27 January 2016).

### 2.1. Characteristics of Participants

Sociodemographic and academic data collected were: gender, age, academic year of study, whether living with parents or not, university courses, existence of financial difficulties, academic pressure (fully bearable, bearable, almost bearable, unbearable, fully unbearable; the latter two responses were grouped together in the analysis in “unbearable” academic pressure), and restful sleep. Academic courses were organized into three groups: healthcare (medicine, pharmacy, physiotherapy, and midwife), university and engineering, and others. Self-reported height and weight were used to calculate body mass index (BMI) using the standard formula (BMI weight [kg]/height [m^2^]) and was classified as: underweight (BMI < 18.5), normal weight (BMI between 18.5 and 24.9), overweight (BMI between 25.0 and 29.9), and obese (BMI > 30) [21].

### 2.2. Screening Questionnaire

Student participants filled in the French five-item “Sick, Control, One stone, Fat, Food” (SCOFF) questionnaire for ED screening [22]. A diagnostic threshold was fixed at two positive responses, with a sensitivity of 0.88 and a specificity of 0.93, using interviews as a diagnostic reference; therefore, data obtained with SCOFF gave a proxy of actual ED [23]. The Expali^TM^-validated algorithmic tool, combining SCOFF and body mass index, was used [21] to screen EDs into three diagnostic categories [24]: restrictive eating disorders, that is, anorexia nervosa, atypical anorexia nervosa, and restrictive food intake disorder; bulimic eating disorders, that is, bulimia nervosa and low-frequency/short-duration bulimia nervosa; and hyperphagic eating disorders, that is, binge-eating disorder and low-frequency/short-duration binge-eating disorder.

The Depression, Stress, and Anxiety Subscales (DASS-21) were used to assess the severity of the core symptoms of depression, anxiety, and stress [24]. We used only the anxiety and stress subscales. The anxiety scale assesses autonomic arousal, skeletal muscle effects, situational anxiety, and the subjective experience of anxious affect. The stress scale is sensitive to levels of chronic non-specific arousal. It assesses difficulty relaxing, nervous arousal, and being easily upset/agitated, irritable/over-reactive and impatient. Each item for each scale is scored from 0 (did not apply to me at all over the previous week) to 3 (applied to me very much or most of the time over the previous week).

### 2.3. Healthcare

Student participants were asked if they had seen a general practitioner (GP) in the previous 12 months, and if yes, their reasons for doing so, including an acute care problem (e.g., pain, influenza), chronic disease (e.g., asthma, diabetes, allergy), emotional problems (anxiety and stress) or prevention patterns (e.g., vaccine, contraception). A visit to the GP, especially due to eating disorders, was not specifically asked. Participants were also asked whether they had already foregone care in the previous 12 months (translation from French: “Have you foregone seeing a doctor?”). If participants answered yes, they had to explain why they had foregone care by answering a multi-part question (i.e., answers were not mutually exclusive) with yes/no answers, including attitudinal barriers, defined as a desire to wait until health improved, preference for self-care with advice from a friend or a website, self-medication, and fear of seeing a doctor; and structural barriers, defined as financial reasons, lack of time, lack of a doctor, and no trust in care.

The resources of help for emotional problems (anxiety and stress) were investigated with a multiple-choice question: “Have you seen a GP, a specialist practitioner, a psychologist, a preventive medicine university practitioner, friends, family, or used the Internet (websites and social networking)?”.

### 2.4. Statistical Analysis

Answers in the online questionnaire were obligatory; thus, no data were missing. Chi-square was used for comparisons of categorical data. Continuous variables were summarized with means, standard deviation (SD), and medians, and compared using the Student t test. All variables with a *p* value < 0.20 were tested in the multivariate analysis. The principal outcome (dependent variables) was the three-category ED measure (restrictive EDs, bulimic EDs, hyperphagic EDs, and no ED as a reference), whereas independent variables were identified by the multivariate analysis with the lower Akaike Information Criterion (AIC), where AIC deals with the trade-off between the goodness-of-fit and the simplicity of the model. The lower AIC included all the variables with a *p* value < 0.20 in the univariate analysis. Interaction terms (i.e., gender*healthcare renunciation, gender*academic pressure, *sleep, gender*academic stress) were tested regarding behavior variables that were included in logistic regression.

The observational study design was approved by the Commission Nationale de l’Informatique et des Libertés (the French Electronic Data Protection Authority) and by Rouen University Hospital’s Institutional Review Board without mandatory informed consent (27 January 2016).

## 3. Results

A total of 1493 college students were included; mean age was 20.1 years (SD = 1.9) and 63.4% were women. Among them, 59.5% were university students, 22.9% healthcare students, and 17.6% engineering students. Sociodemographic characteristics are presented in Table 1.

**Figure 1:**
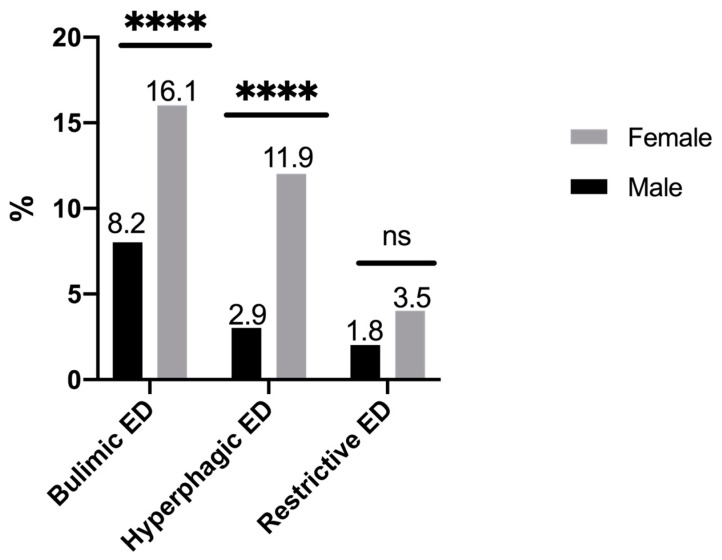
Prevalence of bulimic, hyperphagic, and restrictive eating disorders according to the gender of students (*n* = 1493). ns: non significant. ****: *p* < 0.0001.

Among students, 8.6% were underweight, 12.4% overweight, and 4.6% obese. The prevalence of likely cases of ED was 24.8% (95% CI, 22.6–27.0) with a higher prevalence in female students (31.6%) than in male students (17.0%); *p* < 0.001. Percentage distributions of bulimic EDs, hyperphagic EDs, and restrictive EDs were 13.3%, 8.6%, and 2.9%, respectively. The distribution according to gender is presented in Figure 1.

Women were four times more likely to have hyperphagic EDs than men (AOR CI 95% 4.04 (2.32–7.03)) and twice more likely to have bulimic EDs (AOR CI 95% 2.20 (1.50–3.22)). There was no significant difference in risk of restrictive ED according to gender. Unbearable academic stress was a factor significantly associated with hyperphagic EDs and bulimic EDs. Each diagnostic category of ED was significantly associated with restless sleep (Table 2).

**Figure 2:**
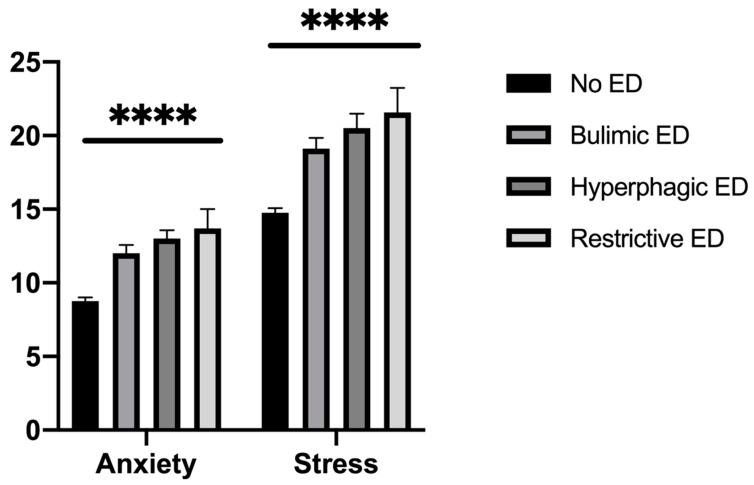
Score of anxiety and stress according to eating disorder (*n* = 1493). ****: *p* < 0.0001.

Courses were not associated with a specific ED diagnostic category. Anxiety and stress levels were higher among students with EDs without a difference between ED diagnostic categories (Figure 2).

**Figure 3:**
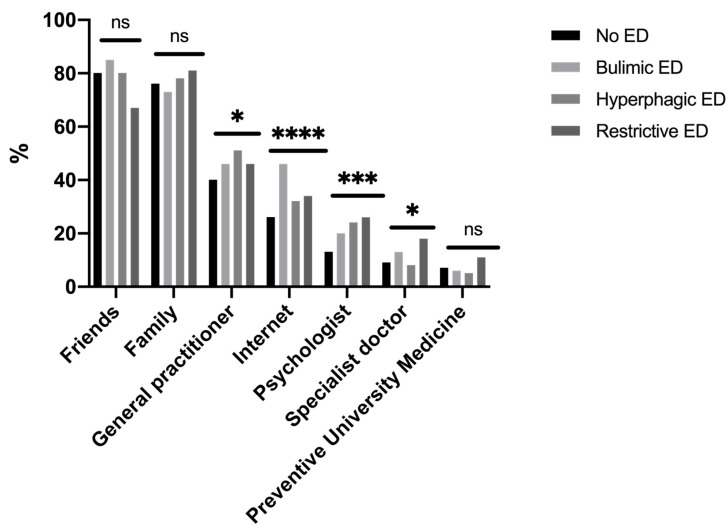
Categories of help-seeking in cases of emotional problems with anxiety and stress (*n* = 1493). ns: non significant. *: *p* < 0.05. **: *p* < 0.01. ***: *p* < 0.001. ****: *p* < 0.0001.

The two main resources for help-seeking in emotional stress situations were friends and family whatever the ED diagnostic category (Figure 3). The third resource was a GP, mostly in students with hyperphagic EDs. Students with bulimic EDs reported using the Internet to search for tips on help. In emotionally stressful situations, students with restrictive EDs sought help from a psychologist or a specialist practitioner.

**Figure 4:**
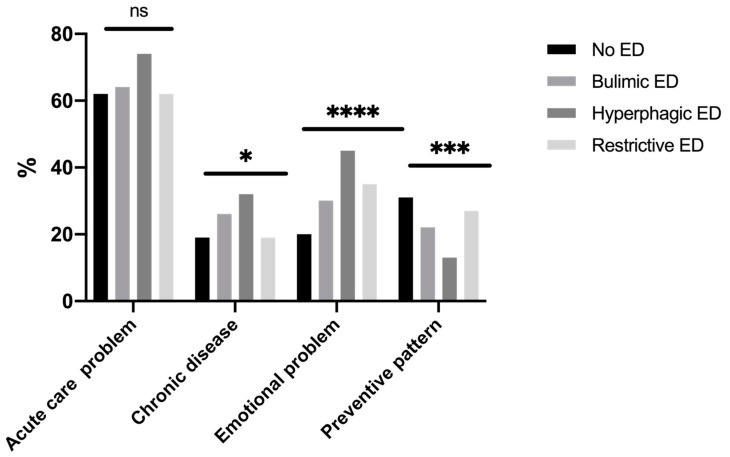
Reasons for foregone care with a general practitioner in the past 12 months (*n* = 1247). ns: non significant. *: *p* < 0.05. ***: *p* < 0.001. ****: *p* < 0.0001.

In the previous 12 months, 84.1% of students had seen their GP at least once without a difference between ED diagnostic categories (Table 1). Reasons for seeing a GP are presented in Figure 4; the main reason given was an acute health problem. Students with EDs (hyperphagic EDs (44.9%), restrictive EDs (35.1%), bulimic EDs (30.2%), and no ED (20.4%) (*p* < 0.0001)) consulted their GP more often for stress or anxiety than students without EDs.

**Figure 5:**
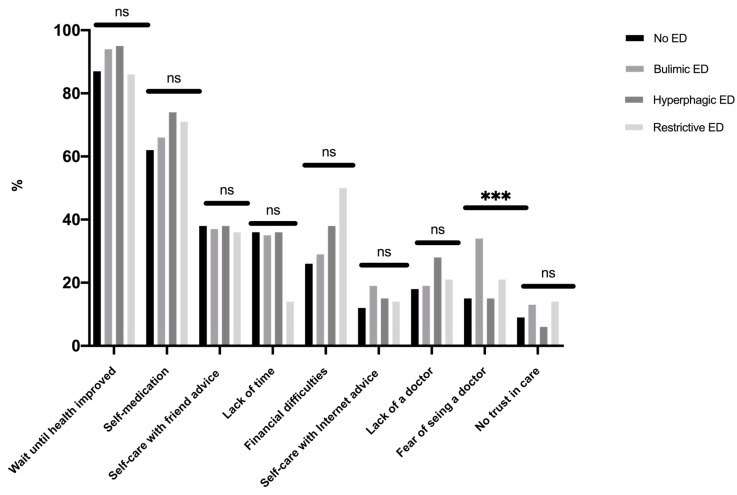
Reasons for foregoing seeing a general practitioner (*n* = 325). ns: non significant. ***: *p* < 0.001.

The prevalence of healthcare renunciation was 21.9% (Table 1). Students with bulimic EDs had a two-fold higher risk of renouncing healthcare than students without EDs (AOR CI 95% 1.91 (1.34–2.72). Reasons for healthcare renunciation are presented in Figure 5; the main reason given was solving the problem on one’s own (waiting until health improved, self-medication, self-care with friends). A financial reason was given by 29.5% of students without differences, according to the ED diagnostic category. The sole reason for healthcare renunciation with a difference between diagnostic categories was a fear of seeing the GP (bulimic EDs 33.8%, restrictive EDs 21.4%, hyperphagic EDs 15.4%, and no ED 15.2% (*p* = 0.008)).

## 4. Discussion

This cross-sectional study, carried out in a convenience sample of a French university, has provided epidemiological data of EDs. Overall, 24.8% of college students were considered likely ED cases, a higher rate than in a previous study in the same university in 2009 (20.5%) [6]. In this student population, the most frequent diagnostic category of ED was bulimic EDs, as in the general population of 18- to 25-year-olds, whereas hyperphagic EDs were more represented in an older population of 18- to 75-year-olds [4]. As expected, EDs were more frequent among women than men, mainly related to hyperphagic EDs [2].

This is the first study conducted in a large population of college students in France, focusing on the relationship between the risk of ED and academic environment and health behavior. Restless sleep was associated with each ED category. Allison et al. showed that insomnia was related to an increased risk of ED, while EDs were related to more disrupted sleep [25]. EDs in young adulthood could be a predictor of sleep disturbances [26]. We found that unbearable academic stress was associated with hyperphagic EDs and bulimic EDs. This association was also reported in another study, though without a distinction between categories of ED [27]. Trigueros et al. reported that academic performance and grading pressure could generate maladaptive consequences for food consumption [28]. Another study found an association between bulimic EDs and hyperphagic EDs with low academic performance, and a higher risk of academic failure among first-year college students [29].

Students with EDs were globally more anxious and stressed than students without EDs. College students in situations of emotional stress (stress and anxiety) were more likely to seek help from friends or family, representing an important step of help-seeking [30]. Attitudes may predict help-seeking intentions related to recognition of symptoms and the benefits of professional help, and openness to treatment for emotional problems [31]. GPs were the third resource in emotional stress situations, especially in students with hyperphagic EDs who reported more recourse to a GP for emotional problems than other students. Improving healthcare professionals’ knowledge at all levels is clearly key to achieving early diagnosis and intervention, but it is equally important to increase awareness among GPs of the clinical, particularly psychological, indicators of EDs [32]. Ali et al. also suggested that help-seeking barriers may differ depending on the type of eating disorder symptomology [33]. The Internet may be useful in seeking help for bulimic EDs. Studies have shown the efficacy of internet- and mobile-based interventions to reduce the consequences of college-related stress, which might potentially attract students who would not otherwise seek help [34] among those with EDs [35,36]. Utz et al. also showed that social networks could help people gain social support from their online networks, which positively affects their well-being [37]. A clinical implication could be the GP’s use of a fast and easy tool (Expali^TM^) combining available data, such as BMI and individual answers to the SCOFF test [24]. Mental health literacy, which refers to one’s knowledge and beliefs about mental disorders, could also be useful for the prevention and early detection of EDs [38,39]. One in five college students renounced healthcare—mostly students with bulimic EDs—with a two-fold risk of that of students without EDs. This higher risk in students with bulimic EDs has also been identified in clinical populations [40]. However, reasons for healthcare renunciation generally did not differ according to the ED diagnostic category. The most common reasons for healthcare renunciation were firstly, attitudinal barriers (e.g., waiting until health improved, self-care), and secondly, structural barriers (e.g., financial reasons, lack of time), consistent with the results of a previous study among students in France [15]. The sole reason with a difference according to ED was a fear of seeing a doctor, concerning mostly bulimic EDs. The shame or guilt of binge-eating could be one explanation for not going to see a GP [41]. Given that the preference to handle problems alone and stigma appear to be critical, there could be a value in determining internet-based psychological treatments, which can be accessed privately and are often built as self-help approaches [42]. A meta-analysis showed a beneficial effect of internet interventions for mental health in university students on global eating disorder symptoms, weight concerns, and affective symptoms [43].

However, caution is advised when generalizing these findings, for the following reasons: First, this study was largely based on self-reported questionnaires; second, it was a convenience sample; and third, this was a cross-sectional study, which did not permit causal relationships to be identified. Voluntary participation could have led to representativeness and self-selection bias. However, the percentage distribution of our convenience sample did not differ from that of the student population of the University of Rouen-Normandy: 59% of women [44], or from that of a European study including 36,000 French students with two-thirds being women [45]. In our sample, the proportion of healthcare students was higher (22%) than in that of the University of Rouen-Normandy (16%), but this bias may have been limited because we previously found that healthcare students did not have a higher risk of ED than the other classes of students [6]. Our sample was a non-clinical population that may have limited information bias because students did not declare an ED when filling out the online questionnaire.

## 5. Conclusions

To the authors’ knowledge, this is the first study conducted in a large population of college students in France, focusing on the relationship between the risk of ED according to diagnostic category, academic environment, and healthcare. One-quarter of students was screened positive for an ED and reported higher levels of academic pressure. Stress management was not necessarily different between students with EDs and students without EDs, but the former had a greater risk of renouncing treatment, especially related to a fear of seeing a GP. GPs should be attentive to student stress, and the need to shift from a weight-focused to a more holistic, individualized, and consistent care approach, with a better balance in targeting psychological and physical problems from an early stage [46]. Emotional intelligence, defined as the ability to perceive, value, and express emotions accurately and to regulate emotions by promoting emotional and intellectual growth has been negatively linked to stress, leading to the generation of EDs [47]. This emotional intelligence could be developed in a student mental health literacy program to better manage help-seeking among college students.

## Figures and Tables

**Table 1:** Characteristics of students according to their eating disorder diagnostic category.

Characteristics	No Eating Disorder (*n* = 1123)	Bulimic EDs (*n* = 198)	Hyperphagic EDs (*n* = 129)	Restrictive EDs (*n* = 43)	Total (*n* = 1493)	*p*
Age mean (SD)	20.1 (1.9)	20.0 (1.9)	20.3 (1.9)	19.8 (1.9)	20.1 (1.9)	0.26
Women (%)	57.7	77.3	87.6	76.7	63.4	<0.0001
Living with parents (%)	69.4	71.2	69.0	65.1	69.5	0.88
Courses (%)						0.003
University	61.8	55.0	46.5	58.1	59.5	
Engineering	17.8	16.7	19.4	11.7	17.6	
Healthcare	20.4	28.3	34.1	30.2	22.9	
Financial difficulties (%)	21.5	31.8	33.3	27.9	24.0	0.001
BMI, kg/m2 mean (SD)	22.0 (3.3)	22.3 (2.8)	26.6 (5.1)	19.5 (2.4)	22.4 (3.7)	<0.0001
Class of BMI (%)						<0.0001
Underweight	9.5	0.0	0.0	51.2	8.6	
Normal weight	77.0	93.0	31.8	48.8	74.4	
Overweight	10.1	4.0	49.6	0.0	12.4	
Obese	3.4	3.0	18.6	0.0	4.6	
Unbearable academic pressure (%)	21.5	34.3	38.8	37.2	25.2	<0.0001
Restless sleep	19.9	31.9	30.6	41.0	23.0	<0.0001
GP visit * (%)	83.4	86.3	86.0	86.0	84.1	0.66
Foregone care * (%)	18.6	34.8	30.2	32.6	21.9	<0.0001

ED: Eating disorders, SD: Standard deviation, BMI: Body Mass Index, *: in the previous 12 months.

**Table 2:** Factors associated with eating disorder diagnostic categories (logistic regression).

Characteristics	No eating Disorder (*n* = 1123)	Bulimic EDs (*n* = 198) AOR (95%CI)	Hyperphagic EDs (*n* = 129) AOR (95%CI)	Restrictive EDs (*n* = 43) AOR (95%CI)
Women	Ref	**2.20 (1.50–3.22)**	**4.04 (2.32–7.03)**	1.83 (0.85–3.95)
Courses	Ref			
University		Ref	Ref	Ref
Engineering		1.11 (0.71–1.74)	1.51 (0.90–2.55)	0.16 (0.02–1.21)
Healthcare		1.14 (0.77–1.68)	1.50 (0.96–2.34)	1.30 (0.63–2.68)
Financial difficulties	Ref	1.29 (0.90–1.84)	1.48 (0.97–2.25)	1.20 (0.58–2.45)
Unbearable academic pressure	Ref	**1.47 (1.03–2.09)**	**1.76 (1.17–2.66)**	1.63 (0.82–3.24)
Restless sleep	Ref	**1.61 (1.12–2.31)**	**1.56 (1.01–2.42)**	**2.22 (1.12–4.40)**
Healthcare renunciation	Ref	**1.91 (1.34–2.72)**	1.36 (0.88–2.10)	1.66 (0.82–3.24)

ED: Eating disorders, AOR: Adjusted Odds Ratio, CI: Confidence Interval, Interaction gender* Healthcare renunciation *p* = 0.75, Interaction gender*academic stress *p* = 0.76, Interaction gender*sleep *p* = 0.64, Interaction gender*course *p* = 0.90. AOR with *p* < 0.05 are in bold.
